# Development of a machine learning model using systemic and ophthalmic parameters to detect sleep-disordered breathing in glaucoma patients

**DOI:** 10.1007/s10384-026-01386-5

**Published:** 2026-06-08

**Authors:** Naoki Kiyota, Mai Yamazaki, Noriko Himori, Takahiro Ninomiya, Koki Osada, Takeshi Yabana, Shigeto Maekawa, Satoru Tsuda, Kazuko Omodaka, Yu Yokoyama, Toru Nakazawa

**Affiliations:** 1 Department of Ophthalmology, Tohoku University Graduate School of Medicine, 1-1 Seiryo-machi, Aoba-ku, Sendai, Miyagi 980-8574 Japan; 2 Seiryo Eye Clinic, Miyagi, Japan; 3 Department of Aging Vision Healthcare, Tohoku University Graduate School of Biomedical Engineering, Miyagi, Japan; 4 Department of Ophthalmic Imaging and Information Analytics, Tohoku University Graduate School of Medicine, Miyagi, Japan; 5 Department of Retinal Disease Control, Tohoku University Graduate School of Medicine, Miyagi, Japan; 6 Department of Advanced Ophthalmic Medicine, Tohoku University Graduate School of Medicine, Miyagi, Japan

**Keywords:** Machine learning, Open-angle glaucoma, Sleep apnea syndrome, Sleep-disordered breathing

## Abstract

**Purpose:**

To develop and validate a machine-learning model using systemic and ophthalmic parameters that predicts sleep-disordered breathing (SDB) in patients with open-angle glaucoma (OAG).

**Study design:**

Retrospective cross-sectional study

**Methods:**

We analyzed 513 patients with OAG (955 eyes) treated at Seiryo Eye Clinic. All the participants underwent comprehensive ophthalmic examinations, including Humphrey visual field (HVF) testing, and home sleep apnea testing (HSAT) to obtain the 4% oxygen desaturation index (ODI). SDB was operationally defined as ODI-SAS, ie, sleep apnea syndrome (SAS) defined by HSAT-derived 4% ODI ≥15 events per hour. Sixteen algorithms were trained to predict ODI-SAS; model performance was assessed by the area under the receiver operating characteristic curve (ROC–AUC), precision, recall, the F1 score, and the Matthews correlation coefficient (MCC).

**Results:**

One hundred fifty-eight patients (30.80%) met the ODI-SAS criterion. ODI-SAS status was associated with a history of hypertension, higher body mass index, family-reported apnea, shorter axial length, fewer antiglaucoma medications, and worse inferocentral total deviation on HVF (all *P* < 0.05), but not with self-awareness of snoring or previous SAS diagnosis. A gradient-boosted decision-tree model (CatBoost) achieved the best performance (ROC–AUC 0.86), with precision 0.692, recall 0.711, F1 score 0.701, and MCC 0.543.

**Conclusion:**

Machine-learning models can predict ODI-SAS in glaucoma using systemic risk factors together with ophthalmic features, including inferocentral visual-field defects. Such models may help identify OAG patients who warrant formal sleep evaluation.

**Supplementary Information:**

The online version contains supplementary material available at https://doi.org/10.1007/s10384-026-01386-5.

## Introduction

Glaucoma features optic nerve head (ONH) cupping and axonal loss of retinal ganglion cells, producing anatomically corresponding visual field (VF) defects. Increased intraocular pressure (IOP) is the only treatable, evidence-based risk factor; however, the Collaborative Normal-Tension Glaucoma Study reported progression in 20% of NTG patients despite well-controlled IOP [1]. This suggests the multifactorial nature of glaucoma, highlighting the need to consider additional risk factors.

Recently, sleep apnea syndrome (SAS) has been recognized as a risk factor for glaucoma development and progression [2–4]. Lin and colleagues analyzed 1012 SAS patients and 6072 controls and provided clear evidence that the 5-year incidence rate of open-angle glaucoma (OAG) was 1.67 times higher in the SAS patients [2]. Kiyota and colleagues found that ONH blood flow might be affected by SAS and that it was related to corresponding VF defects in OAG patients [3]. Yamada and colleagues found that oxidative stress was significantly higher in glaucoma patients with SAS than in glaucoma patients without SAS and that VF defects progressed faster in the SAS group [4]. Thus, treating SAS might also have the benefit of making glaucoma progression milder. Nevertheless, we first need to identify patients with undetected SAS complications to determine this. Interestingly, while 2% to 27% [5, 6] of SAS patients are reported to have glaucoma, 20% to 78% [7, 8] of glaucoma patients are found to have SAS, with the latter rate being higher. This highlights the importance of ophthalmologists considering whether glaucoma patients might have SAS and of referring them for proper testing.

In recent years, machine learning has advanced across predictive tasks, with powerful models such as support vector machines (SVMs), random forests, and neural networks. For SAS, machine learning has been applied to diagnosis and severity prediction with ~90% accuracy [9, 10]. However, these studies largely used data from internal-medicine populations—especially sleep clinics—and relied on features such as neck circumference, Mallampati classification, electrocardiography (ECG), and oxygen saturation, which are not routinely collected in eye clinics. The high SAS prevalence in sleep-clinic cohorts also introduces selection bias, limiting generalizability. Consequently, applying those models directly to glaucoma patients is challenging. A glaucoma-specific model is therefore needed, developed by screening SAS within a glaucoma population.

Polysomnography (PSG) confirms SAS by measuring the apnea-hypopnea index (AHI), the number of apneas and hypopneas per sleep hour. PSG is costly and labor-intensive and often requires an overnight clinic stay; COVID-19 restrictions further limited access, underscoring these challenges. As an alternative, home sleep apnea testing (HSAT) uses a simple home device to monitor breathing, oxygen saturation, and other sleep parameters [11]. The 4% oxygen desaturation index (ODI) counts ≥4% drops in saturation per hour and correlates well with PSG-measured AHI, and ≥15 events/hour is commonly used to indicate SAS [12]. Because HSAT for all glaucoma patients is impractical, targeted HSAT for those strongly suspected of having SAS is appropriate, but clear criteria to flag SAS comorbidity in glaucoma are still lacking.

Inspired by our recent findings that inferocentral VF defects are linked to SAS [3, 13], we hypothesized that using ophthalmic parameters, including inferocentral total deviation (TD) measured with Humphrey visual-field (HVF) testing, might enhance prediction of SAS comorbidity in glaucoma patients. Considering the recent advancements in machine learning in the field of medicine [14], we hypothesized that a machine learning model incorporating both systemic and ophthalmic parameters could effectively predict SAS comorbidity. Here, we compared 16 different machine learning models and found that the CatBoost model exhibited the highest average area under the curve (AUC) at 0.80, with the best AUC reaching 0.86, making it the most effective model for predicting 4% ODI-SAS in glaucoma patients.

## Patients and methods

### Patients

This retrospective observational study comprised 950 patients who visited the Seiryo Eye Clinic in Miyagi Prefecture and underwent HSAT from June 2020 to November 2022. At their initial visit to our hospital, each patient underwent a medical history review that included hypertension, diabetes mellitus, dyslipidemia, heart disease, migraine, thyroid disease, cold extremities, cancer, SAS, and smoking history. This review also involved asking about family-reported apnea and the patients’ self-awareness of snoring, and daytime sleepiness was assessed by use of the Epworth Sleepiness Scale (ESS).

**Fig. 1:**
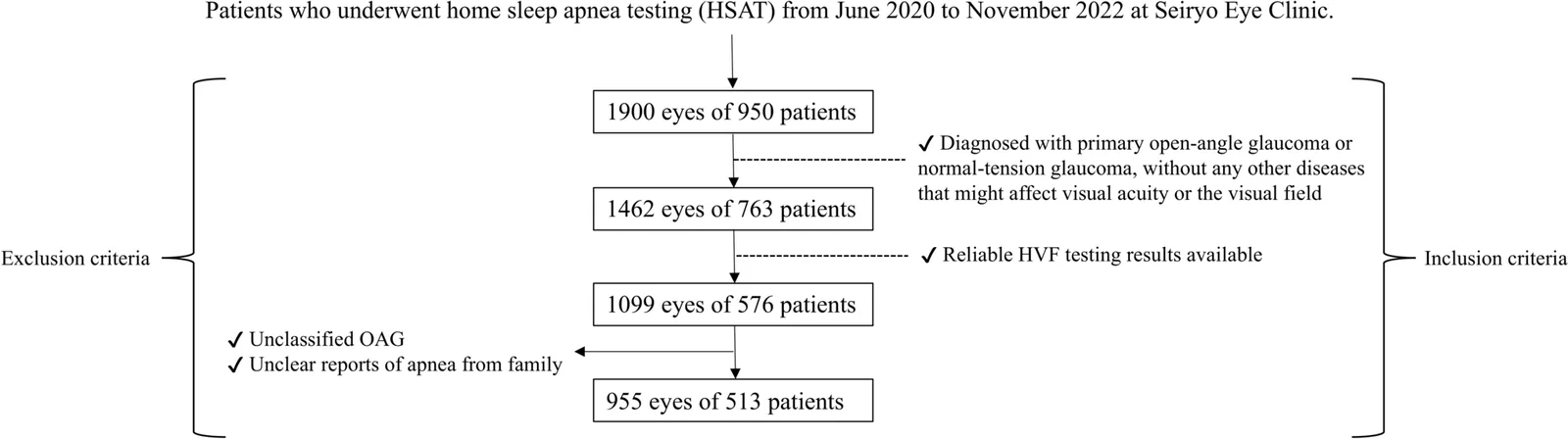
Flow of eye selection in this study. The leftward horizontal arrows denote the exclusion criteria, and the dotted line on the right denotes the inclusion criteria

Figure 1 provides details of the selection process. A glaucoma specialist identified 1462 eyes of 763 patients as having OAG, which included primary OAG, NTG, and unclassified OAG. Diagnoses were based on the presence of an open angle on gonioscopy findings, untreated IOP, and the presence of glaucomatous changes in the optic disc with corresponding VF defects that satisfied the Anderson-Patella criteria [15]. None of the eyes diagnosed with OAG had received glaucoma surgery, nor did they exhibit any other diseases that might potentially impact the VF (eg, corneal opacity; clinically evident cataract [lens nucleus grade >2 in the Emery-Little classification]; retinal diseases, including macular degeneration and retinal vascular diseases, optic neuritis, and brain infarction) [13, 16]. Reliable VF test data were available for 1099 eyes of 576 patients. Eyes with OAG that was unclassified owing to a lack of data on untreated IOP, and patients who could not be accurately parameterized owing to unclear family reports of apnea, were excluded. Consequently, a total of 955 eyes from 513 patients were included in the final statistical analysis and machine learning models. To further ensure diagnostic accuracy, the diagnoses of the 513 patients (955 eyes) included in the final analysis were rereviewed by a glaucoma specialist (M.Y.), and no discrepancies were identified. If both eyes of a patient met the inclusion criteria, both eyes were included in the analysis.

The procedures of this study were approved by the institutional review board of Tohoku University Graduate School of Medicine (study no. 2022-1-732) and conducted in accordance with the principles of the Declaration of Helsinki. Written informed consent was obtained from all the participants.

### Measurement of clinical variables

We performed a complete ophthalmologic examination of each patient, which included measuring best-corrected visual acuity (logarithm of the minimum angle of resolution [logMAR]) and axial length; performing fundoscopy, a slit-lamp examination, and gonioscopy; using Goldmann applanation tonometry to measure IOP; using the Humphrey Field Analyzer (HFA; Carl Zeiss) to measure mean deviation (MD) and TD, which indicates how much a patient’s visual sensitivity deviates from average values, in each measurement point; and evaluating the optic disc with a 90-D lens. A glaucoma specialist performed all the examinations. Central corneal thickness was measured by use of anterior-segment optical coherence tomography (OCT; CASIA, Tomey Corporation). Axial length was measured with the OA-2000 (Tomey Corporation), and circumpapillary retinal nerve fiber layer thickness (cpRNFLT) was measured by use of DRI-OCT (Triton; Topcon). The accompanying software was used to divide the peripapillary area into the superior, temporal, inferior, and nasal sectors; cpRNFLT was measured in each sector.

**Fig. 2:**
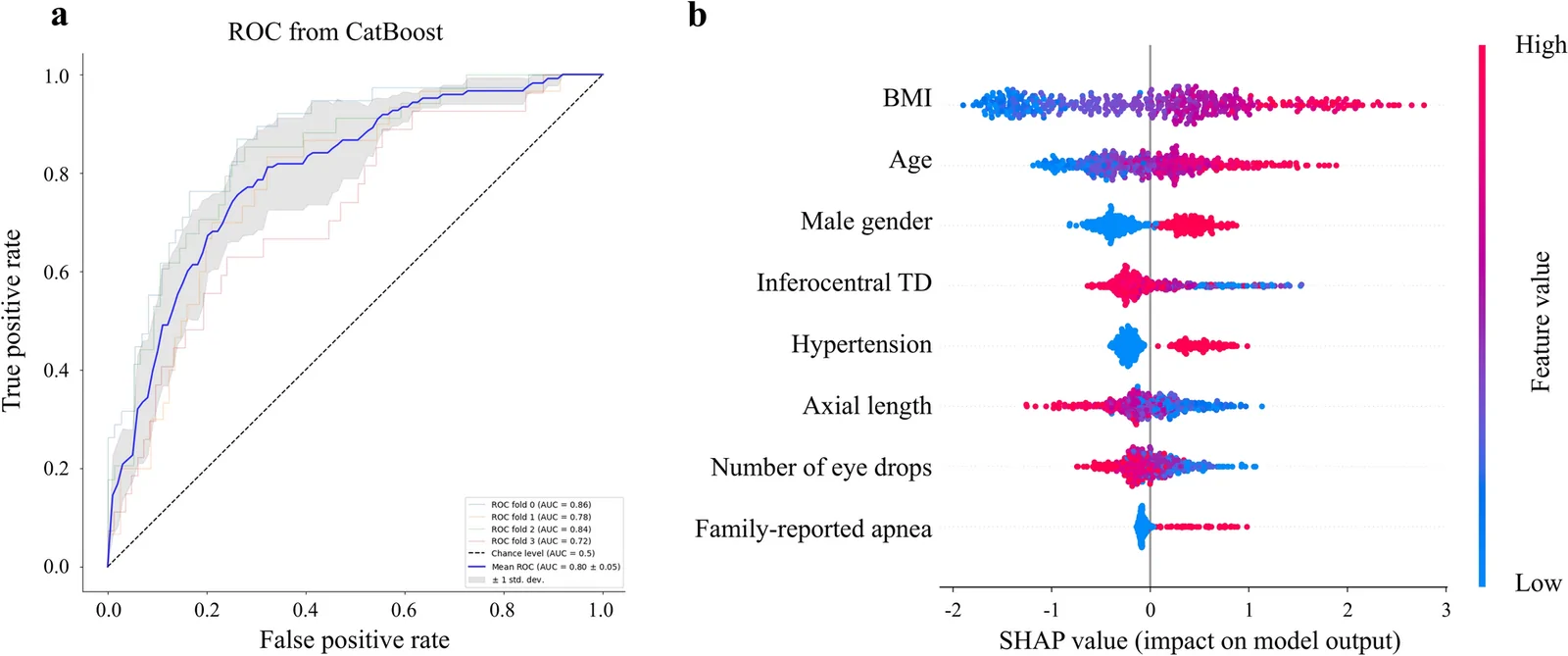
Representative images of both eyes in a patient with open-angle glaucoma (OAG). **a**–**e** Right eye: **a** visual-field (VF) sector map measured with the Humphrey Field Analyzer (HFA) 24-2 Swedish Interactive Threshold Algorithm (SITA); **b** VF grayscale (HFA 24-2 SITA); **c** retinal nerve fiber layer (RNFL) thickness by clock-hour sectors; **d** laser speckle flowgraphy (LSFG) color map; **e** fundus photograph of the optic disc. **f**–**j** Left eye: **f** VF sector map (HFA 24-2 SITA); **g** VF grayscale (HFA 24-2 SITA); **h** RNFL thickness by clock-hour sectors; **i** LSFG color map; **j** fundus photograph of the optic disc. S, I, N, and T denote superior, inferior, nasal, and temporal, respectively

The VF was measured by use of the standard 24-2 Swedish interactive threshold algorithm program of the HFA. Reliable HVF testing data in this study corresponded to data with fixation errors <20%, false positives <33%, and false negatives <33% [17, 18]. The site-specific VF was evaluated with reference to the modified version of the VF sector map established by Garway-Heath and colleagues [19]. Since the VF sector corresponding to the nasal ONH (the gray region in Fig. 2) is less important in glaucoma [20], this study focused on the superior, central, and inferior VF sectors (the red, orange, and blue regions, respectively, in Fig. 2). The anatomic correspondence between TD and OCT-measured cpRNFLT has previously been described [3]. The central 6 measurement points were further divided into the superocentral quadrant (the upper 3 points) and the inferocentral quadrant (the lower 3 points) [13]. We calculated the mean TD in each sector.

ONH blood flow was measured by use of laser speckle flowgraphy (LSFG; Softcare Corporation). The main measurement parameter was the mean blur rate (MBR), from which was derived the tissue-area MBR (MT); this was calculated as the average MBR within the tissue area of the ONH. The MT has been reported to represent blood flow in the deep areas of the ONH [21, 22]. Before the patients underwent fundus imaging, they received 0.4% tropicamide (Mydrin M; Santen Pharmaceutical), to dilate their pupils. After instillation, the patients sat in a dark, quiet room for 15 minutes to stabilize pupil dilation. Blood pressure and pulse rate were then measured with the HBP-1300 (Omron).

The method for measuring oxidative stress in this study is routinely used [23]. All blood analyses were performed with a free radical analyzer system (Free Carpe Diem, Wismerll Company) equipped with a spectrophotometer reader, a thermostatically controlled mini centrifuge, and a measurement kit. The analyses were performed according to the manufacturer’s instructions. Blood samples were collected at least 3 hours after the last meal. Oxidative stress was measured by obtaining serum samples and administering the diacron-reactive oxygen metabolites (dROMs) test, which quantitatively measures total peroxides, including hydroperoxides. The results were expressed in Carratelli units (U. CARR). Total antioxidants in the serum were determined by use of a biologic antioxidant potential (BAP) assay. Total antioxidant capacity in the serum was determined by quantifying the reduction to iron. The results were expressed as µmol/L of reduced Fe^3+^.

### Measurement of 4% ODI using the HSAT device

The HSAT device used in this study was the SAS-2100 (Nihon Kohden Corporation), which measures pressure changes via a nasal cannula placed under the nose. The device records nasal respiratory pressure flow, snoring waveforms, oxygen saturation, and pulse rate during sleep. From these signals, the device automatically outputs several indices, including the HSAT-AHI and the 4% oxygen desaturation index (4% ODI), which are calculated independently as separate measures from the same overnight recording. Since total sleep time cannot be measured by HSAT, the AHI must be calculated on the basis of the total recording time. Therefore, HSAT has a limited capacity to calculate the true AHI, and as described, we adopted the 4% ODI to evaluate SAS on the basis of previous reports that used HSAT [12]. Because PSG-derived AHI was available only in a subset of the participants, restricting model development to PSG-performed cases would reduce the sample size and could introduce selection bias; therefore, we used HSAT-derived 4% ODI-defined ODI-SAS as the primary outcome. We divided the OAG patients into groups on the basis of the presence or absence of ODI-SAS, as indicated by ≥15 4% ODI events/hour, following previous reports [12].

### Statistical analysis

All the data were expressed as means ± standard deviations. When clinical data from the day of the SAS test were unavailable, we used the clinical data obtained closest to the SAS test date within a 3-month window.

First, we used data from 513 eyes of 513 patients to perform linear regression analysis with 4% ODI-SAS as the explanatory variable (0 indicated non-4% ODI-SAS and 1 indicated 4% ODI-SAS) and systemic factors as response variables to determine the effect of being in the 4% ODI-SAS group on each parameter. For variables that were significant on univariable linear regression analysis, we performed multivariable linear regression analysis adjusted for age and sex (0 indicated female and 1 indicated male; Table 1). We conducted a subanalysis in participants with both PSG-derived AHI and the 4% ODI available (*n* = 231) and evaluated their association by use of the Pearson correlation coefficient.

**Table 1:** Differences in systemic clinical characteristics between the non-4% ODI-SAS and 4% ODI-SAS groups

Variables	Non-4% ODI-SAS	4% ODI-SAS	*P* value	Adjusted *P* value
	*n* = 355	*n* = 158		
Age, y	59.7 ± 12.0	65.6 ± 11.1	<0.001*	–
Male sex, n (%)	146 (41.13%)	100 (63.29%)	<0.001*	–
Body mass index, kg/m^2^	22.09 ± 3.31	24.86 ± 3.19	<0.001*	<0.001*
Smoking, n (%)	40 (11.27%)	18 (11.39%)	0.984	0.816
Hypertension, n (%)	93 (26.20%)	79 (50.00%)	<0.001*	<0.001*
Diabetes mellitus, n (%)	38 (10.70%)	30 (18.99%)	0.011*	0.157
Dyslipidemia, n (%)	158 (44.51%)	88 (55.70%)	0.016*	0.100
Heart disease, n (%)	55 (15.19%)	27 (17.09%)	0.649	0.407
Migraine, n (%)	68 (19.15%)	15 (9.49%)	0.006*	0.259
Thyroid disease, n (%)	18 (5.07%)	5 (3.16%)	0.282	0.718
Cold extremities, n (%)	138 (38.87%)	41 (25.95%)	0.005*	0.134
Cancer history, n (%)	37 (10.42%)	17 (10.76%)	0.909	0.248
Systolic BP, mm Hg	123.88 ± 19.59	131.93 ± 17.54	<0.001*	0.011*
Diastolic BP, mm Hg	77.51 ± 12.49	81.87 ± 11.14	<0.001*	0.003*
Pulse rate, bpm	68.05 ± 11.05	69.22 ± 11.22	0.375	0.199
HSAT AHI, events/h	11.56 ± 7.17	31.59 ± 12.06	<0.001*	<0.001*
4% ODI, events/h	7.55 ± 3.85	25.52 ± 10.10	–	–
dROMs, U. CARR	376.03 ± 68.14	373.38 ± 56.89	0.963	0.648
BAP, μmol/L	2038.86 ± 272.76	1932.82 ± 306.76	<0.001*	<0.001*
ESS score	7.87 ± 4.52	7.34 ± 4.67	0.219	0.815
Self-awareness of snoring, n (%)	117 (32.96%)	57 (36.08%)	0.518	0.172
Apnea noted by family, n (%)	37 (10.42%)	38 (24.05%)	<0.001*	0.002^*^
SAS history, n (%)	29 (8.17%)	24 (15.19%)	0.016*	0.091

*AHI* apnea-hypopnea index, *BP* blood pressure, *ODI* oxygen desaturation index, *dROMs* diacron reactive oxygen metabolites,

*BAP* biologic antioxidant potential, *ESS* Epworth Sleepiness Scale, *SAS* sleep apnea syndrome, *U. CARR* Carratelli units

*P* values represent the effect of having 4% ODI-SAS on each systemic parameter on linear regression analysis.

Adjusted *P* values indicate the effect, adjusted for age and sex, on multivariable linear regression analysis.

^*^indicates significance.

Next, we used data from 955 eyes of 513 patients for an ocular parameter analysis. We employed a linear mixed-effects model with 4% ODI-SAS as the explanatory variable, each ocular parameter as a response variable, and the participant variable as the random effect. For parameters significant in this univariable linear mixed-effects model, we used a multivariable linear mixed-effects model with adjustments for age and sex (Table 2). These analyses were conducted with R software (version 4.1.3), with the significance level set at *P* <0.05. We considered age, sex, and parameters significantly different between the groups after age and sex adjustments as potential candidates for predicting 4% ODI-SAS in the machine learning training. We did not adopt some parameters in consideration of their lack of generalizability and to maintain the simplicity of the model for potential clinical application in the future. For hypertension history and systolic/diastolic blood pressure, we opted for hypertension history, as it is simpler to inquire about in a questionnaire, and blood pressure values are subject to variability and treatment intervention. Adding BAP to the best AUC model, the CatBoost model, as described below, slightly increased the AUC from 0.86 to 0.87, but BAP was not adopted in the model in this study to ensure the examination’s generalizability. Temporal cpRNFLT and inferocentral TD can be considered related parameters owing to their anatomic correspondence. We adopted inferocentral TD rather than temporal cpRNFLT because the HVF test is likely to be already performed in glaucoma diagnosis, whereas OCT requires consideration of device differences. In addition, we have recently reported the association of inferocentral TD with SAS [13]. Thus, age, sex, body mass index (BMI), hypertension, family-reported apnea, inferocentral TD, number of eye drops, and axial length were selected as explanatory variables for the subsequent machine learning model training.

**Table 2:** Differences in ocular characteristics between the non-4% ODI-SAS and 4% ODI-SAS groups

Variables	Non-4% ODI-SAS *n* = 665	4% ODI-SAS *n* = 290	*P* value	Adjusted *P* value
BCVA, logMAR	0.01 ± 0.26	0.07 ± 0.31	0.002^*^	0.087
IOP, mm Hg	12.81 ± 3.43	11.88 ± 2.58	0.050	0.203
CCT, μm	512.78 ± 33.24	510.53 ± 37.70	0.606	0.806
Axial length, mm	25.76 ± 1.89	25.18 ± 1.82	0.003^*^	0.008^*^
CpRNFLT, μm	66.48 ± 17.05	62.64 ± 18.31	0.009^*^	0.185
CpRNFLT-superior, μm	78.86 ± 25.13	73.94 ± 26.78	0.026^*^	0.422
CpRNFLT-temporal, μm	61.07 ± 20.81	54.28 ± 19.27	<0.001^*^	0.014^*^
CpRNFLT-inferior, μm	70.00 ± 27.30	66.96 ± 25.85	0.180	0.883
MT-overall, AU	9.23 ± 2.60	8.25 ± 2.52	<0.001^*^	0.108
MT-superior, AU	9.62 ± 3.12	8.64 ± 2.97	0.002^*^	0.338
MT-temporal, AU	7.33 ± 2.53	6.41 ± 2.39	<0.001^*^	0.074
MT-inferior, AU	9.67 ± 2.89	8.62 ± 2.73	<0.001^*^	0.081
Number of eye drops, n	3.45 ± 1.40	3.32 ± 1.36	0.358	0.043^*^
MD, dB	−10.95 ± 8.10	−12.33 ± 8.79	0.055	0.790
TD-superior, dB	−12.35 ± 10.44	−13.08 ± 10.61	0.461	0.706
TD-superocentral, dB	−11.83 ± 11.78	−12.69 ± 12.14	0.387	0.601
TD-inferocentral, dB	−6.66 ± 9.11	−9.65 ± 11.29	<0.001^*^	0.020^*^
TD-inferior, dB	−10.67 ± 9.35	−12.41 ± 10.29	0.033^*^	0.584

*BCVA* best-corrected visual acuity, *logMAR* logarithm of the minimum angle of resolution, *IOP* intraocular pressure,

*CCT* central corneal thickness, *cpRNFLT* circumpapillary retinal nerve fiber layer thickness, *MT* tissue-area mean blur rate,

*MD* mean deviation, *TD* average total deviation, *AU* arbitrary units, *SAS* sleep apnea syndrome

*P* values represent the effect of having 4% ODI-SAS on each ocular parameter on linear mixed-effects analysis.

Adjusted *P* values indicate this effect, adjusted for age and sex, on multivariable linear mixed-effects analysis.

^*^indicates significance.

### Machine learning models

We developed binary classification models with 16 different machine learning algorithms and compared their performance on the basis of the AUCs. The algorithms included extreme gradient boosting (XGBoost), random forest, extra trees, light gradient boosting machine (LightGBM), CatBoost, AdaBoost, gradient boosting, bagging, histogram-based gradient boosting, SVM, logistic regression, decision tree, K-nearest neighbors (KNN), Gaussian naive Bayes, ridge classifier, and lasso. Explanations of these models were provided in previous reports [24–27].

As shown later, CatBoost showed the best AUC in this study. CatBoost is a high-performance gradient boosting framework specifically designed to handle categorical features without extensive preprocessing, such as one-hot encoding. Introduced by Yandex, CatBoost uses ordered boosting and a combination of random permutations to reduce target leakage and overfitting, making it especially powerful for datasets with many categorical features. For CatBoost and all other models used, default parameters were applied on the basis of previous studies [24–26], as extensive hyperparameter tuning was not feasible in this setting owing to the relatively small dataset size, making grid search or other optimization methods impractical.

Four-fold cross-validation was conducted to evaluate each algorithm’s accuracy, with only data from the right eye of each patient being used to avoid potential bias from including both eyes of a single patient. To address class imbalance, we applied the synthetic minority over-sampling technique (SMOTE) during the training phase, balancing class representation by generating synthetic examples of the minority class. This approach minimized overfitting risks associated with the majority class, supporting a more robust training process. For model interpretability, we used Shapley additive explanations (SHAP) to analyze each feature’s contribution to the model’s predictions. A SHAP beeswarm plot was used to visualize the impact of various features on the model’s decision-making process, providing insight into the most influential features across the different algorithms.

All machine learning analyses were conducted with Python 3.8.15. Numeric operations were handled by NumPy version 1.23.5, and data manipulation was performed with Pandas version 2.0.3. Data visualization was facilitated by Seaborn version 0.12.2. Machine learning algorithms were implemented by use of Scikit-learn version 1.3.0, with boosting algorithms handled by XGBoost version 1.7.6, LightGBM version 4.1.0, and CatBoost version 1.2.5. To address class imbalance, Imbalanced-learn version 0.12.2 was used, and model interpretation was conducted by use of SHAP version 0.42.1. All the analyses were conducted on an Apple M1 8-core CPU that included 4 performance cores and 4 efficiency cores, running macOS Sonoma 14.4.1 with 8 GB of unified memory.

## Results

Table 1 shows the difference in systemic clinical characteristics between the 4% ODI-SAS and non-4% ODI-SAS groups. The 4% ODI-SAS group included 158/513 (30.80%) patients. On univariable analysis, age (*P* < 0.001), male sex (*P* < 0.001), BMI (*P* < 0.001), hypertension (*P* < 0.001), diabetes mellitus (*P* = 0.011), dyslipidemia (*P* = 0.016), migraine (*P* = 0.006), cold extremities (*P* = 0.005), systolic blood pressure (*P* < 0.001), diastolic blood pressure (*P* < 0001), BAP (*P* < 0001), family-reported history of apnea (*P* < 0.001), and history of SAS (*P* = .016) significantly differed between the groups. After the age and sex adjustment, significant differences between the 2 groups were found in BMI (*P* < 0.001), hypertension (*P* < 0.001), systolic blood pressure (*P* = .011), diastolic blood pressure (*P* = 0.003), BAP (*P* < 0.001), and family-reported history of apnea (*P* = 0.002). The difference between the groups in SAS history, self-awareness of snoring, and ESS score did not reach statistical significance (*P* = 0.091, *P* = 0.172, and *P* = 0.815, respectively). Thus, systemic parameters such as older age, male sex, obesity, hypertension, and oxidative stress were associated with ODI-SAS comorbidity. In a subanalysis of participants with both the PSG-AHI and the 4% ODI available (*n* = 231), the 4% ODI showed a positive correlation with the PSG-AHI (Pearson r = 0.562, *P* < 0.001).

Table 2 shows the differences in the ocular characteristics of the groups. In the univariable linear mixed-effects model, best-corrected visual acuity (*P* = 0.002), axial length (*P* = 0.003), cpRNFLT (*P* = 0.009), superior cpRNFLT (*P* = 0.026), temporal cpRNFLT (*P* < 0.001), MT overall (*P* < 0.001), superior MT (*P* = 0.002), temporal MT (*P* < 0.001), inferior MT (*P* < 0.001), inferocentral TD on VF testing (*P* < 0.001), and inferior TD on VF testing (*P* = 0.033) differed significantly. In the multivariable linear mixed-effects model, after adjustment for age and sex, axial length was shorter (*P* = 0.008), temporal cpRNFLT was lower (*P* = 0.014), number of eye drops was lower (*P* = 0.043), and inferocentral TD was lower (*P* = 0.020) in the 4% ODI-SAS group. Shorter axial length, a lower number of glaucoma eye drops, and neurodegeneration on the temporal side of the ONH, which corresponds to inferocentral VF defects, were associated with ODI-SAS comorbidity.

**Fig. 3:**
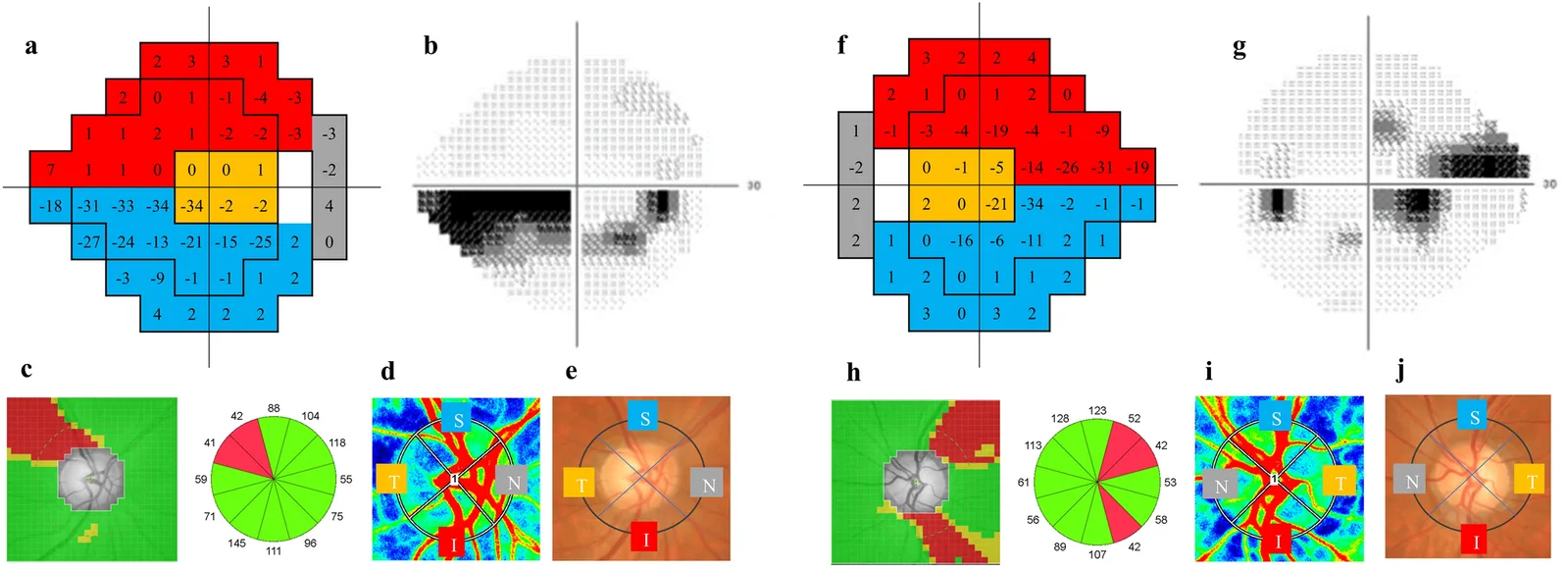
Receiver operating characteristic (ROC) analysis and SHAP interpretation of the CatBoost model. **a** ROC curves for the 4 cross-validation folds (colored lines) and the mean ROC (blue); the shaded area indicates ±1 SD, and the diagonal dashed line denotes chance level. **b** Shapley additive explanations (SHAP) beeswarm plot. Features are ordered by decreasing mean absolute SHAP value. Each point represents 1 eye; the x-position is the SHAP value for that feature (positive values push the prediction toward ODI-SAS), and the color encodes the feature value from low (blue) to high (red)

CatBoost exhibited the highest average AUC, followed by random forest and Gaussian naive Bayes, with values of 0.802 ± 0.053, 0.797 ± 0.044, and 0.794 ± 0.033, respectively, as shown in Supplementary Table S1. The receiver operating characteristic (ROC) curve for each fold of CatBoost is displayed in Figure 3A. The best AUC for CatBoost was 0.864 (fold 0). In this best-performing model, the precision was 0.692 and the recall was 0.711, resulting in an F1 score of 0.701 and a Matthews correlation coefficient of 0.543. This AUC is comparable to those of previously reported machine learning models that used many internal-medicine parameters, such as neck circumference, ECG, and oxygen saturation, among people attending sleep clinics [9, 10]. If we remove the ocular parameters from our CatBoost model, the average AUC decreases from 0.802 to 0.763, and from 0.864 to 0.783 in the best model, indicating the importance of ocular parameters to achieve this high AUC and to predict the 4% ODI-SAS group.

Figure 3B presents the results for SHAP values and feature importance plots in the best CatBoost model, graphically depicting the contribution of each feature variable to the prediction of 4% ODI-SAS as the objective variable. The x-axis shows the SHAP value (negative to positive impact on the prediction of 4% ODI-SAS), and the y-axis lists the features. Color encodes the feature value (low = blue, high = red). Factors with a wider distribution on the horizontal axis are considered important for predicting 4% ODI-SAS, and the arrangement from top to bottom corresponds to their order of contribution. Whilst BMI and age had the highest mean SHAP values, these parameters displayed many purple points, indicating significant variations in feature values. In contrast, inferocentral TD, with the fourth-highest mean SHAP value, showed relatively clearer separations based on feature values compared with BMI or age. Moreover, it demonstrated contributions to the model that surpassed some systemic factors, such as a history of hypertension or a report of apnea from a family member. These findings indicate that in addition to known systemic factors, machine learning that incorporates ophthalmic parameters from routine glaucoma examinations can predict 4% ODI-SAS status with high accuracy.

## Discussion

SAS likely contributes to glaucoma pathophysiology, yet predicting SAS in glaucoma remains difficult, complicating collaboration with internists. In our cohort, untreated SAS was present in ~30% of OAG patients, emphasizing the need for case-finding. Self-reported snoring and previous SAS diagnosis did not identify undetected SAS. A higher incidence of ≥15 4% ODI events/hour was linked to male sex and older age. After age/sex adjustment, the 4% ODI-SAS group showed higher BMI and hypertension, elevated systolic/diastolic pressures, lower BAP, more family-reported apnea, reduced temporal cpRNFLT, lower inferocentral TD, fewer eye drops, and shorter axial length. We used age, sex, BMI, hypertension, family-reported apnea, inferocentral TD, number of eye drops, and axial length to train 16 machine learning models; CatBoost performed best (ROC–AUC 0.86).

ODI-SAS prevalence was 30.80%, with the remaining 69.20% classified as non–ODI-SAS, consistent with previous reports of 20% to 78% SAS prevalence among glaucoma patients [7, 8]. Self-awareness of snoring was ~30% in both groups, substantially lower than rates reported in working men (42.3% with AHI <15 events/hour and 72% with AHI ≥15 events/hour) [28], possibly because our survey was conducted in an eye-clinic context where patients did not anticipate sleep-related questions. Taken together, conventional symptom-based items such as snoring awareness and ESS may not reliably identify ODI-SAS in an OAG clinic population, underscoring the value of a complementary machine-learning approach using routinely available clinical data, including ophthalmic parameters.

Antioxidant capacity was lower in the ODI-SAS group, consistent with previous reports of reduced antioxidant capacity in SAS/OSA [29, 30]. Oxidative stress—an imbalance between oxidant production and antioxidant defenses—may promote inflammation, sympathetic activation, cytotoxicity, and lipid oxidation, contributing to vascular endothelial damage, atherosclerosis, hypertension, and heart failure [31], and may also damage retinal ganglion cells, potentially linking SAS/OSA to glaucoma pathophysiology. To maintain model simplicity and generalizability, we did not include BAP in the final machine-learning model, although it could improve prediction if this assay becomes more widely available in clinical practice.

In this study, patients in the ODI-SAS group were more likely to have a history of hypertension and showed lower values for ONH-tissue blood flow as measured by LSFG. As the multivariable linear mixed-effects model with age and sex as explanatory variables showed no statistical difference in MT, we cannot rule out the possibility that the difference in the MT parameters was due to differences in age and sex. However, we have previously demonstrated that at least among healthy Japanese individuals, age and sex did not affect MT [32]. Taken together, these findings suggest that the presence of ODI-SAS may be associated with lower MT, although residual confounding by age and sex cannot be excluded. In line with this, Lin and colleagues reported significantly reduced LSFG-derived MBR parameters (MA, MV, and MT) in patients with severe OSA (PSG-AHI ≥30) compared with those with nonsevere OSA (PSG-AHI <30) and noted that MA/MV/MT remained significantly lower after sex adjustment, whereas between-group differences in waveform parameters were no longer significant [33]. Although direct comparison is limited by differences in the outcome definition (PSG-AHI-based OSA vs HSAT-derived 4% ODI-based ODI-SAS) and study population (general/nonglaucoma vs glaucoma), these observations together support the possibility that OSA/SDB may influence ONH microcirculation. Consistent with the vascular comorbidity of OSA/SDB, hypertension was more prevalent in the ODI-SAS group and was also the fifth-highest contributor in the machine learning model.

Interpreting the link between fewer eye drops, shorter axial length, and SAS is challenging. Postsurgical eyes were excluded, and IOP and glaucoma severity did not differ between the SAS and non-SAS groups. Fewer drops in the SAS group suggest comparable IOP control with less medication, implicating non-IOP mechanisms of neurodegeneration. Because longer axial length (myopia) increases glaucoma risk [34], myopic eyes may dilute the relative contribution of SAS—consistent with the shorter axial length observed in the 4% ODI-SAS group. Even without a direct pathologic link, including axial length in the machine learning models helps adjust for such background bias.

Most importantly, we developed a machine learning model and achieved a high AUC (0.864) for predicting 4% ODI-SAS. Previously, attempts to predict the severity of SAS by using an SVM resulted in an AUC of only 0.65 [35], which was not considered satisfactory. Rowley and colleagues employed 4 clinical prediction formulas to predict a PSG-measured AHI of >10 events/hour and >20 events/hour, achieving an AUC range of 0.669–0.757 [36], which also appears to be insufficient. Some studies have achieved higher AUCs (0.87–0.929) by using parameters that include SAS-specific parameters [37], blood tests [38], or computed tomography scans [39], which, however, generally require significant time and effort, especially for ophthalmologists. Our model is primarily based on parameters acquired through patient interviews and general glaucoma care, conveniently allowing for the prediction of SAS without additional effort. We are exploring the development of an application or medical program based on our findings to facilitate the easier prediction of SAS in the clinic in the future.

CatBoost is a gradient-boosted decision-tree algorithm notable for handling categorical data without one-hot or label encoding. This avoids feature-space inflation and spurious bias from ad hoc encodings and reduces the preprocessing burden. In medical datasets—where categorical variables such as sex, hypertension status, and SAS indication are common—this capability likely contributed to the favorable performance of CatBoost relative to the other tree algorithms. CatBoost also employs ordered boosting with random permutations to limit target leakage and mitigate overfitting, which is advantageous for our relatively small dataset. Together, these properties improved model robustness in our analyses.

This study has several limitations. First, it was cross-sectional and lacks continuous HVF data, so SAS risk may be underestimated in eyes that currently lack inferocentral VF defects but may develop them later; longitudinal follow-up—particularly slopes of inferocentral TD—could improve prediction. Second, despite the advantages of HSAT, its self-administered nature sometimes yields insufficient or inaccurate recordings. Our ultimate goal is to predict SAS as defined by PSG-measured AHI, and we plan to collect such PSG data. Nevertheless, the ability to predict SAS from the HSAT-derived 4% ODI already has practical value because no clear criteria exist for even suspecting SAS in glaucoma patients in routine clinical care, and targeted screening is warranted. Accordingly, this model should be interpreted as an adjunctive tool to help identify OAG patients who may warrant HSAT and, when indicated, further definitive evaluation including PSG, rather than as a substitute for PSG-based diagnosis. Third, model accuracy was constrained by the limited sample size. Ideally, extensive hyperparameter tuning (eg, grid search) would improve performance, but the dataset size restricted meaningful gains. Likewise, using separate datasets for feature selection, tuning, and cross-validation was not feasible. Because the model was tailored to glaucoma patients, spectrum/generalizability biases may remain, limiting applicability to other eye diseases, healthy populations, or different glaucoma cohorts. Nevertheless, previous work links male sex [40], obesity [41], and inferocentral VF defects [13] to SAS, supporting external relevance. Future studies should expand cohorts and enable more robust optimization and validation. Fourth, because ODI-SAS was operationally defined by using a single HSAT-derived 4% ODI cutoff (≥15 events/h) [12], some participants with mild-to-borderline ODI values below this threshold (eg, 5–14 events/h) may have been classified as non–ODI-SAS, which could have attenuated between-group differences and biased associations toward the null. Nonetheless, we selected the ≥15 events/h threshold because it has been widely used in previous HSAT-based studies and systematic reviews as a clinically meaningful cutoff to identify at least moderate SDB/OSA, and it aligns with our objective to capture clinically relevant disease. Therefore, our findings should be interpreted as relating to ODI-defined, at least moderate SDB rather than to PSG-defined disease severity across the full spectrum. Despite these limitations, this remains one of the largest studies to examine ODI-SAS specifically in glaucoma.

In conclusion, a CatBoost model combining ocular parameters (including inferocentral TD) with systemic risk factors achieved an ROC–AUC 0.86 for predicting 4% ODI–SAS. Such a model may help ophthalmologists detect suspected ODI-SAS earlier and refer patients promptly, potentially improving both overall and glaucoma-specific outcomes.

## Supplementary Information

Below is the link to the electronic supplementary material.

Supplementary file1 (DOCX 23 kb)
