# Supplementary material for: Development of a machine learning model using systemic and ophthalmic parameters to detect sleep-disordered breathing in glaucoma patients
Source: Jpn J Ophthalmol. 2026 Jun 8;70(5):1218–28. doi: 10.1007/s10384-026-01386-5 (PMC13615987; doi:10.1007/s10384-026-01386-5)
Supplement: Supplementary file 1 [file 10384_2026_1386_MOESM1_ESM.docx]

| **Supplementary Table S1** Performance comparison of machine learning algorithms for SAS detection | | | | | | | |
| --- | --- | --- | --- | --- | --- | --- | --- |
|  | | | | | | | |
| Models | ROC-AUC | Precision | Recall | Accuracy | Specificity | F1 | MCC |
| CatBoost | 0.802 ± 0.053 | 0.568 ± 0.085 | 0.645 ± 0.072 | 0.756 ± 0.024 | 0.800 ± 0.033 | 0.601 ± 0.072 | 0.430 ± 0.078 |
| Random forest | 0.797 ± 0.044 | 0.567 ± 0.076 | 0.603 ± 0.080 | 0.751 ± 0.014 | 0.812 ± 0.033 | 0.581 ± 0.062 | 0.408 ± 0.062 |
| Gaussian naive Bayes | 0.794 ± 0.033 | 0.515 ± 0.060 | 0.727 ± 0.105 | 0.722 ± 0.014 | 0.719 ± 0.047 | 0.598 ± 0.058 | 0.414 ± 0.059 |
| Ridge classifier | 0.791 ± 0.015 | 0.520 ± 0.063 | 0.737 ± 0.028 | 0.722 ± 0.033 | 0.716 ± 0.054 | 0.606 ± 0.040 | 0.419 ± 0.037 |
| Lasso | 0.791 ± 0.016 | 0.520 ± 0.063 | 0.737 ± 0.028 | 0.722 ± 0.033 | 0.716 ± 0.054 | 0.606 ± 0.040 | 0.419 ± 0.037 |
| Extra trees | 0.790 ± 0.031 | 0.559 ± 0.085 | 0.550 ± 0.044 | 0.740 ± 0.030 | 0.819 ± 0.044 | 0.551 ± 0.054 | 0.371 ± 0.066 |
| Logistic | 0.790 ± 0.015 | 0.522 ± 0.054 | 0.712 ± 0.045 | 0.726 ± 0.017 | 0.732 ± 0.037 | 0.600 ± 0.040 | 0.412 ± 0.027 |
| Histogram-based gradient boosting | 0.785 ± 0.040 | 0.555 ± 0.064 | 0.637 ± 0.123 | 0.751 ± 0.022 | 0.795 ± 0.019 | 0.590 ± 0.088 | 0.416 ± 0.094 |
| Gradient boosting | 0.769 ± 0.033 | 0.516 ± 0.058 | 0.65 ± 0.093 | 0.722 ± 0.020 | 0.751 ± 0.037 | 0.571 ± 0.059 | 0.377 ± 0.063 |
| eXtreme Gradient Boosting | 0.767 ± 0.052 | 0.495 ± 0.081 | 0.541 ± 0.095 | 0.710 ± 0.022 | 0.776 ± 0.030 | 0.515 ± 0.084 | 0.310 ± 0.085 |
| Support vector machine | 0.759 ± 0.009 | 0.473 ± 0.036 | 0.757 ± 0.033 | 0.686 ± 0.022 | 0.653 ± 0.038 | 0.582 ± 0.037 | 0.374 ± 0.039 |
| Light gradient boosting machine | 0.757 ± 0.047 | 0.538 ± 0.109 | 0.594 ± 0.088 | 0.733 ± 0.040 | 0.791 ± 0.048 | 0.560 ± 0.087 | 0.375 ± 0.109 |
| k-Nearest neighbors | 0.751 ± 0.038 | 0.492 ± 0.085 | 0.648 ± 0.059 | 0.699 ± 0.044 | 0.720 ± 0.063 | 0.555 ± 0.068 | 0.346 ± 0.077 |
| Bagging | 0.745 ± 0.049 | 0.493 ± 0.078 | 0.625 ± 0.024 | 0.701 ± 0.033 | 0.733 ± 0.042 | 0.549 ± 0.059 | 0.338 ± 0.066 |
| AdaBoost | 0.741 ± 0.029 | 0.498 ± 0.075 | 0.634 ± 0.120 | 0.708 ± 0.019 | 0.739 ± 0.048 | 0.552 ± 0.076 | 0.352 ± 0.078 |
| Decision tree | 0.622 ± 0.031 | 0.427 ± 0.078 | 0.549 ± 0.040 | 0.652 ± 0.027 | 0.695 ± 0.037 | 0.478 ± 0.059 | 0.230 ± 0.065 |
|  | | | | | | | |
| *ROC-AUC* receiver operating characteristic area under the curve, *MCC* Matthews correlation coefficient | | | | | | | |
